# Methuselah’s daughters: Paternal age has little effect on offspring number and quality in *Cardiocondyla* ants

**DOI:** 10.1002/ece3.4666

**Published:** 2018-11-08

**Authors:** Jürgen Heinze, Michaela Hanoeffner, Jacques H. C. Delabie, Alexandra Schrempf

**Affiliations:** ^1^ LS Zoologie/Evolutionsbiologie Universität Regensburg Regensburg Germany; ^2^ Myrmecological Laboratory Cocoa Research Center CEPLAC Ilhéus Bahia Brazil; ^3^ Santa Cruz State University‐DCAA Ilhéus Bahia Brazil

**Keywords:** ants, offspring weight, paternal age, reproductive success, sperm quality

## Abstract

Male age may directly or indirectly affect the fitness of their female mating partners and their joint progeny. While in some taxa of insects, old males make better mates and fathers, young males excel in others. Males of most social Hymenoptera are relatively short lived and because of testis degeneration have only a limited sperm supply. In contrast, the wingless fighter males of the ant *Cardiocondyla obscurior* live for several weeks and produce sperm throughout their lives. Wingless males engage in lethal combat with rival males and the winner of such fights can monopolize mating with all female sexuals that emerge in their nests over a prolonged timespan. Here, we investigate if male age has an influence on sperm quality, the queen's lifespan and productivity, and the size and weight of their offspring. Queens mated to one‐week or six‐week‐old males did not differ in life expectancy and offspring production, but the daughters of young males were slightly heavier than those of old males. Our data suggest negligible reproductive senescence of *C. obscurior* males even at an age, which only few of them reach. This matches the reproductive strategy of *Cardiocondyla* ants, in which freshly emerging female sexuals rarely have the option to mate with males other than the one present in their natal nest.

## 1 INTRODUCTION

The age of a potential mating partner might constitute an important trait on which female choice is based. On the one hand, old age might indicate high viability and genetic quality and old males might therefore be preferred (Kokko, 1998; Kokko & Lindström, 1996; Manning, 1985). On the other hand, the accumulation of negative germline mutations (Beck & Promislow, 2007; Radwan, 2003) and damaged sperm (Reinhardt & Siva‐Jothy, 2005; Siva‐Jothy, 2000) might make old males inferior mates.

Empirical studies in insects have yielded ambiguous results about the relationship between male quality and age, suggesting that it varies widely among species. For example, sperm viability increased with age in the cricket *Teleogryllus oceanicus* (García‐González & Simmons, 2005), the mates of old males lived longer in the chrysomelid beetle *Ophraella communa* (Zhao et al., 2017) and were more fecund in *Drosophila pseudoobscura* (Avent, Price, & Wedell, 2008), and the offspring of old *Drosophila bipectinata* males showed a higher mating ability and life span (Krishna, Santhosh, & Hegde, 2012). In contrast, older males of *D. montana* achieved fewer matings (Hoikkala, Saarikettu, Kotiaho, & Lümatainen, 2008), and the fecundity of females of *D. melanogaster* decreased with the age of their mating partners (Koppik & Fricke, 2017; Ruhmann, Koppik, Wolfner, & Fricke, 2018). In the hide beetle *Dermestes maculatus* and the cabbage beetle, *Colaphellus bowringi*, males of intermediate age achieved more matings and their partners had the highest fecundity (Hale, Elgar, & Jones, 2008; Jones, Featherston, Paris, & Elgar, 2007; Liu, Xu, He, Kuang, & Xue, 2011). Finally, male age had no effect on various fitness parameters in the seed beetle *Callosobruchus maculatus* (Fricke & Maklakov, 2007) and did not relate with mating success in the European corn borer, *Ostrinia nubilalis* (Milonas & Andow, 2010).

Males of many Hymenoptera are sperm limited, because their testes degenerate early in adult life (Boivin, Jacob, & Damiens, 2005; Boomsma, Baer, & Heinze, 2005; Heinze, 2016). Consequently, males transfer fewer and fewer sperm during successive copulations (e.g., Ramadan, Wong, & Wong, 1991; Damiens & Boivin, 2005), and even in species with sperm replenishment, females that mated with old males often lay a larger percentage of unfertilized eggs than females that mated with young males (He & Wang, 2008; Srivastava & Singh, 1995). Because of haplodiploid sex determination, unfertilized eggs develop into males, so that sperm‐depleted females produce a male‐biased sex ratio (e.g., Godfray, 1990; King, 2000). Furthermore, in honey bees, a species in which males can mate only once, sperm number and viability were found to decrease with increasing male age (Locke & Peng, 1993; Mazeed & Mohanny, 2010; Stürup, Baer‐Imhoff, Nash, Boomsma, & Baer, 2013; but see Rousseau, Fournier, & Giovenazzo, 2015). Old Hymenopteran males therefore appear to generally make poor partners.

The wingless (“ergatoid”) males of the ant genus *Cardiocondyla* are exceptional as they are the only known males of social Hymenoptera with lifelong spermatogenesis (Heinze & Hölldobler, 1993). Wingless males of many tropical species of *Cardiocondyla* engage in lethal fighting with rival males in their natal nest (Heinze, 2017; Kinomura & Yamauchi, 1987; Stuart, Francoeur, & Loiselle, 1987). The winners of such fights may monopolize mating with all female sexuals emerging over many weeks. Here, we examine how male age affects queen fitness as well as the size and weight of the queen's first offspring. Given that colony fitness depends on the performance of the present male, we expected only a weak influence even of very old males on the fecundity and longevity of the queen and the quality of their offspring. Using two different age classes of queens in our experiment, we at the same time determined how a prolonged premating period affects reproductive performance.

## 2 MATERIALS AND METHODS

### 2.1 Study species and experimental setup

Experimental colonies of *Cardiocondyla obscurior* Wheeler 1929 were set up with 20 workers and five larvae each from well‐established stock colonies that originally derive from colonies collected in 2009 in an introduced population at Ilhéus, Bahia, Brazil. Female sexuals and wingless, “ergatoid” males of *Cardiocondyla* readily mate under laboratory conditions, usually within a week after emergence. To obtain mating between sexuals of different age classes, each experimental colony received either one female sexual pupa (Y) or a four‐week‐old virgin female sexual (mature, M), and either one male pupa (Y) or a six‐week‐old unmated male (O). Mated queens of *C. obscurior* may live for more than 30 weeks (Oettler & Schrempf, 2016). Four‐week‐old queens therefore can still be considered as “young,” even though they would have mated long before if a male had been present. Even without mating, virgin queens may shed their wings within a few weeks after emergence and begin to mature eggs (Schrempf, Heinze, & Cremer, 2005; see Schmidt, Frohschammer, Schrempf, & Heinze, 2014 for mother–son mating in a related species). Nevertheless, because a long premating time reduces the period during which queens can lay fertilized eggs (e.g., Fuester, Swan, Taylor, & Ramaseshiah, 2008), we expected a small negative influence of prolonged virginity on reproductive performance. Males live considerably shorter than queens, and only around 10% reach an age of six weeks or more (Metzler, Heinze, & Schrempf, 2016).

We aimed to have 12 replicates for each cross, but due to the failure of pupae emerging and the availability of sexuals, we had 12 experimental colonies with both sexuals being young (YY), 10 with a mature queen and an old male (MO), 11 with a mature queen and a young male (MY), and 10 with a young queen and an old male (YO). Colonies were kept in incubators with 12‐hr 24°C/12‐hr 25°C temperature cycles and were fed three times per week with honey and cockroaches or fruit flies ad libitum. The number of workers was standardized weekly to 20 by removing or adding individuals from stock colonies, as we wanted to avoid any influence of colony size on fitness parameters.

### 2.2 Age dependency of sperm traits

Sperm viability was studied by dissecting the seminal vesicles of five old and five young males in Beadle solution (128.3 mM NaCl, 4.7 mM KCl, 2.3 mM CaCl_2_) and staining sperm with 5 µl of SYBR‐14 working solution from a LIVE/DEAD Cell‐Mediated Cytotoxicity Kit (L‐7011, SYBR stock solution diluted 1:50 in Beadle solution, Thermo Fischer Scientific, Waltham, MA, USA) in the dark for 10 min. Thereafter, 2 µl of propidium iodide was added, carefully mixed and incubated for further 7 min in the dark. Live (green) and dead (red) sperm cells were determined by fluorescent microscopy (Axiophot, Carl Zeiss, Oberkochen, Germany, magnification 200×). Sperm viability could not be determined in one of the five old males.

To determine sperm length, we measured 10–40 sperm cells each from ten young and ten old males using a dark field microscope at 200× magnification and the software analysis 2.1 (total 684 sperm cells, DigiVision Pro 2.10.100; burster, Gernsbach, Germany). Measurement error, determined by ten times measuring sperm from one individual, was <1.5%.

### 2.3 Effects of male age on queen fitness and offspring traits

We monitored the timing of wing loss and the begin of egg laying by queens, their life span, weekly egg laying rate, and total sexual production. The hatchability of eggs was determined by comparing the total number of pupae by the sum of eggs counted per week. It takes about one week for an egg to develop into a larva (Schrempf & Heinze, 2006), so eggs may have been counted twice and the absolute value of hatchability is therefore probably underestimated. Nevertheless, results can be compared across colonies as the same procedure was used in all crosses.

The first emerging workers (two to six, depending on availability) and all female sexual and male offspring from each queen were frozen at −20°C. We determined fresh weight of 1–6 workers per colony using an SC2 microbalance (Sartorius, Göttingen, Germany) and thereafter mounted them for morphometry. Head width (across the eyes), thorax length (Weber's length), and the area of the left and the right eye were measured at 200× magnification using a Keyence VHX‐500FD digital microscope (VHX‐500FD, Keyence, Osaka, Japan). All measures were determined in µm. The measurement error, obtained by ten times weighing or measuring the same individual, was <1%. Fluctuating asymmetry was determined as the difference between the areas of left and right eye.

All data are given in Appendix S1.

### 2.4 Statistics

We investigated the impact of queen and male age on different traits by generalized linear models (GLMs) using R (R Development Core Team, 2015), starting with a full model (*x* = male age + queen age + male age: queen age) and removing factors without a significant influence. For comparisons of weight, size, and fluctuating asymmetry among the offspring of queens, we included “mother queen” as a random factor in a generalized linear mixed model (GLMMs). When necessary, data were transformed to approach normal distribution and homogenize variances (see Appendix S2). Differences concerning mating success, life expectancy of queens, sperm viability, and sperm length were analyzed using parametric and nonparametric tests in Statistica 6.0 (StatSoft, Tulsa, OK, USA) and Past 3.07 (Hammer, Harper, & Ryan, 2001). As a quantitative measure for the magnitude of the observed differences, we estimated effect sizes (Cohen's *d*) online at https://www.psychometrica.de/effect_size.html based on the results of *t* tests, ANOVA, and Mann–Whitney *U* tests. Because the same data sets were used in different tests, differences in queen traits (mean egg production, start of egg production, wing shedding, total sexuals, sex ratio, queen bias, egg hatchability) are significant at level of 0.05 only at *p* < 0.0036, for worker offspring traits (thorax length, head width, fluctuating asymmetry) at *p* < 0.0083.

## 3 RESULTS

### 3.1 Age dependency of sperm traits

**Figure 1:**
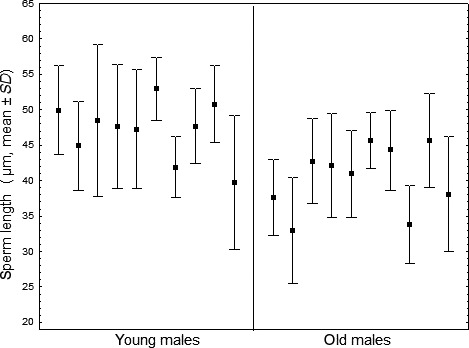
Sperm length of ten young (1 week old) and ten old (6 weeks old) wingless males of the ant *Cardiocondyla obscurior*. Sperm length differs between the two age classes (ANOVA, *p* < 0.0001). Note that the *y*‐axis does not start at zero

Sperm viability was generally high and did not differ between young and old males (proportion of dead sperm, young males, *N* = 5, median 0.020, old males, *N* = 4, median 0.019, Mann–Whitney *U* test, *U* = 9, *p* = 0.806, effect size *d*_Cohen_ = 0.164; see also Metzler, Schrempf, & Heinze, 2018). Mean sperm length differed among males (ANOVA; *df* = 19, *F* = 23.85, *d*_Cohen_ = 2.302; *p* < 0.0001) and young males had significantly longer sperm than old males (mean of mean lengths of spermatozoa from 10 young males, 47.14 µm ± *SD* 4.01 µm, old males 40.41 ± *SD* 4.62 µm, *t*‐test, *df* = 18, *t* = 3.477, *p* = 0.003; *d*_Cohen_ = 1.555; Figure 1).

### 3.2 Effects of male age on queen fitness and offspring traits

Sexuals mated in only 25 of the 43 experimental setups. Young males appeared to be more successful than old males (16 of 23, 69.6% vs. 9 of 20, 45%), although the difference was not significant (Fisher's exact test, *p* = 0.130). Queen age did not have an influence on mating success (13 of 22, 59% young queens and 12 of 21, 57%, mature queens mated, Fisher's exact test, *p* = 1.00). Offspring was obtained from nine of 12 YY colonies (75%), five of 10 MO colonies (50%), seven of 11 MY colonies (64%), and four of 10 YO colonies (40%).

Male age (16 young, 9 old) did not influence queen life span (log rank test, WW = 0.256, *p* = 0.907). Neither queen nor male age had a significant influence on weekly egg lying rate, total sexual production (number of all sexuals produced), sex ratio (female sexuals/all sexuals; only 11 young and 6 old queens produced sexuals), and queen bias (female sexuals/total female offspring) (GLMs, *p* > 0.18 for queen age, male age, and interaction; see Appendix S2; Mann–Whitney *U* tests, all *p* > 0.05; see Appendix S3). Queen age had an effect on the timing of wing shedding, in that all but one mature queens had already shed their wings before the experimental colonies were established and a male was added while queens that had been added as pupae shed their wings only after the addition of the male (GLM, *p* < 0.00001 for queen age, significant at *p* < 0.01 after Bonferroni's correction; male age and interaction *p* > 0.05; *U* = 9.5, *p* = 0.00004, *d*_Cohen_ = 2.235, see Appendix S2 and S3). Similarly, queen age appeared to have a slight effect on the start of egg laying after colony setup (GLM, *p* = 0.068 for queen age, male age and interaction *p* > 0.05, *U* = 44.5, *p* = 0.068, *d*_Cohen_ = 0.783, see Appendix S2 and S3).

Egg hatchability (pupae/eggs) varied strongly among colonies. Male age appeared to have a weak but insignificant influence on hatching success, with eggs laid by queens mated to a young male yielding more pupae (GLM, *p* = 0.078 for male age; queen age and interaction *p* > 0.05; male age: *U* = 38, *p* = 0.057, *d*_Cohen_ = 0.83, see Appendix S2 and S3).

Worker offspring of young and mature queens appeared to differ in thorax length (35 daughters of young queens, 28 daughters of mature queens; GLMM, *p* = 0.017 for queen age and *p* = 0.051 for the interaction between male and queen age, after removal of the nonsignificant interaction *p* > 0.05; queen age: *U* = 389, *p* = 0.166, *d*_Cohen_ = 0.358), but neither head width (GLMMs, *p* > 0.05 for queen age, male age, and interaction; *U* = 352.5, *p* = 0.157) nor fluctuating asymmetry of eye area varied with parental age (GLMMs, *p* > 0.05 for queen age, male age, and interaction; Mann–Whitney *U* tests, all *p* > 0.05, see Appendix S2 and S3).

**Figure 2:**
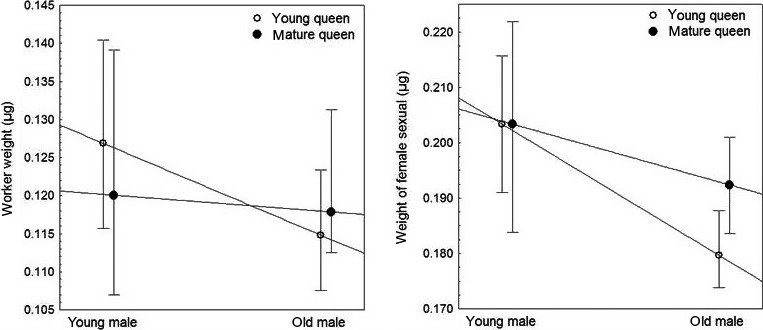
The effect of male age (1 vs. 6 weeks) on body weight (median, quartiles) of freshly emerged workers and female sexuals produced by young (1 week) or mature queens (4 weeks) of the ant *Cardiocondyla obscurior*. The offspring sired by young males is larger than progeny of old males, (GLMM, workers: *p* = 0.0058; female sexuals: *p* = 0.0009; interaction between queen age and male age *p* > 0.05). Note that the *y*‐axis does not start at zero

Worker daughters of young males were significantly heavier than daughters of old males (GLMM, *p* = 0.0058 for male age, significant at *p* < 0.05 after Bonferroni's correction; queen age and interaction *p* > 0.05; *U* = 1,148, *p* = 0.009, *d*_Cohen_ = 0.493; see Appendix S2 and S3, Figure 2). Weighing female sexuals showed the same result, that is, daughters of young males were heavier than daughters of old males (GLMM, *p* = 0.0009 for male age, significant at *p* < 0.01 after Bonferroni's correction; queen age and interaction *p* > 0.05, *U* = 362, *p* = 0.001, *d*_Cohen_ = 0.664; see Appendix S2 and S3, Figure 2). Examining the influence of male age on the weight of workers and female sexuals across the four different mating types in detail revealed that the offspring of YY queens was significantly larger than that of YO queens (Kruskal–Wallis test and pairwise Mann–Whitney *U* tests with Bonferroni's correction: workers: *H* = 8.716, *p* = 0.0331, YY vs. YO: *U* = 239.5, *p*_corr_ = 0.027; female sexuals: *H* = 11.11, *p* = 0.0112; YY vs. YO: *U* = 100, *p*_corr_ = 0.020, all other pairwise comparisons *p*_corr_ > 0.1, Figure 2). Nevertheless, the GLMMs did not reveal any statistically significant interaction at *p* < 0.05. The small number of males produced by old queens did not allow a meaningful statistical analysis (young, *N* = 19, 0.136, 0.126, 0.142, old, *N* = 3, range 0.127 – 0.137).

## 4 DISCUSSION

In many animals, parental age has a strong effect on reproductive performance and the fitness of their offspring. This does not appear to be the case in the ant *Cardiocondyla obscurior*. A prolonged premating period of queens (4 weeks vs. 1 week) did not visibly affect their productivity and the traits of their offspring, except that most 4‐week‐old queens had already shed their wings before mating and laid their first eggs faster than young queens. Similarly, a comparison between queens mated with either 1‐week or 6‐week‐old wingless males did not reveal a clear influence of male age on the longevity and productivity of the queens. The mating success of old males and the hatchability of eggs fertilized by their sperm appeared to be lower than the corresponding values of young males (at *p*‐values of 0.130 and 0.078, respectively) and a larger sample size may have yielded a significant difference. Nevertheless, in GLMs, the mates of old and young males were indistinguishable in weekly egg laying rate, total sexual production, sex ratio, and queen bias at *p*‐values > 0.18. Hence, even at an age, which is reached only by 10% of them, *C. obscurior* males apparently suffer little from reproductive senescence. This is also evident from the analysis of sperm viability of males of different ages (see also Metzler et al., 2018). The only clearly significant effects of male age were that young males had on average longer sperm than old males and that the daughters of young males were significantly heavier than the daughters of old males.

In contrast to most other species of social insects, sexuals of *Cardiocondyla* mate in their natal nest. Males of *C. obscurior* and other species monopolize mating with all emerging female sexuals by eliminating most rival males shortly after emergence when the latter are still relatively helpless and have a soft, unsclerotized cuticula (Heinze, 2017; Kinomura & Yamauchi, 1987; Stuart et al., 1987). Young males are steadily produced in the colonies, but because of this competitive asymmetry adult males usually succeed in remaining the only male in a colony for weeks or, in a few species, even for many months (Yamauchi, Ishida, Hashim, & Heinze, 2006). Female sexuals of *C. obscurior* appear to mate only once (Schmidt, Trindl, Schrempf, & Heinze, 2016) and normally do not have a choice with whom to mate. The fitness of the colony therefore depends strongly on the quality of the present male. This special reproductive biology may have led to the evolution of prolonged spermatogenesis (Heinze & Hölldobler, 1993) and, as we show here, negligible reproductive senescence in males.

While in our study, younger males produced on average longer sperm than old males, Metzler et al. (2018) could not substantiate any age effect on sperm length. However, in our study, males were not allowed to mate before the start of the experiment, which might mean that sperm cells in their seminal vesicles were relatively old. In contrast, in the study by Metzler et al. (2018), old males had regular access to female sexuals and constantly replenished their sperm supplies. Our result might therefore more reflect the effect of sperm age on sperm length. In any case, varying sperm length did not influence queen productivity. While sperm aging has been shown to negatively affect offspring fitness (e.g., Pizzari, Dean, Pacey, Moore, & Bonsall, 2008), sperm length and male fitness are not consistently correlated (e.g., Simmons, Wernham, García‐González, & Kamien, 2003). In perennial social insects, such as ants, sperm is much longer stored in the queen's spermatheca than in the males' seminal vesicles and therefore might be relatively insensitive to aging (e.g., Stürup et al., 2013).

Both worker and female sexual offspring of young males were significantly heavier than the daughters of old males, with an intermediate effect size. The evolutionary importance of this difference remains unclear. In female sexuals, weight shortly after emergence does not necessarily reflect weight at maturity, as young queens may fatten before mating (e.g., Keller & Passera, 1988; Keller & Ross, 1993; Martin, 1991). Furthermore, female sexuals of *C. obscurior* rarely disperse and found new colonies solitarily but instead are usually accompanied by workers if they leave their natal nest at all (e.g., Heinze & Delabie, 2005). Hence, for female sexuals weight after eclosion presumably has little effect on dispersal capabilities and founding success. Whether worker fresh weight at eclosion has an influence on their behavior, for example, concerning division of labor remains to be studied. At least fat content has been shown to affect division of labor in social insects, with foragers being generally less fat than nurses (e.g., Blanchard, Orledge, Reynolds, & Franks, 2000; Toth, Kantarovich, Meisel, & Robinson, 2005; Bernadou, Busch, & Heinze, 2015).

How male age proximately affects the weight of their offspring is presently unknown. Paternal effects on offspring quality are known from a number of insects in which males provide their mates with nutrition, for example, nuptial gifts or large spermatophores, or in which they directly engage in brood care (e.g., Crean & Bonduriansky, 2014). In other cases, offspring traits may reflect the response of female physiology to varying composition of male seminal fluids. For example, in the ladybird beetle, *Cheilomenes sexmaculatus*, both daughters and sons of older males tended to be heavier than those of younger fathers (Mirhosseini, Michaud, Jalali, & Ziaaddini, 2014), probably because old males transfer more or qualitatively different seminal fluids (Obata, 1987). In *Drosophila melanogaster*, like in *C. obscurio*r, the hatchability of eggs decreased slightly with male age. Interestingly, this decline paralleled the decreasing expression of genes coding for several seminal fluid proteins known to elicit postmating changes in the female (Koppik & Fricke, 2017). In *C. obscurior*, the effect of male age appeared to be more pronounced in the offspring of young queens, perhaps indicating that mature queens are less sensitive to variation in male traits.

In social insects, workers rather than the queens nourish and groom the brood, which provides additional complexity (e.g., Linksvayer, 2006). In *C. obscurior*, in addition to male age also the age of nurse workers and the age of the queen (one week vs. 12 weeks) appear to influence the size of offspring workers (Giehr, Heinze, & Schrempf, 2017), indicating that the age distribution both of the breeding pair and the complete colony may impact the provisioning of the brood and thus offspring traits.

Our study again highlights the flexibility of reproductive strategies and in particular their interrelation with aging in social insects and nicely complements previous reports on the lack of reproductive senescence in *Cardiocondyla* queens (e.g., Oettler & Schrempf, 2016). With their lifelong sperm production, wingless *Cardiocondyla* males are unique among the ~20,000 species of social ants, bees, and wasps, and how they proximately maintain functioning testes and high sperm quality over weeks needs to be further explored.

## CONFLICT OF INTEREST

None declared.

## AUTHOR CONTRIBUTIONS

JH and AS designed experiment, JHCD helped collecting the ants, MH did laboratory experiments, JH analyzed data and wrote the manuscript.

## DATA ACCESSIBILITY

Raw data from this study are included as Appendix S1.
