# Supplementary material for: Methuselah’s daughters: Paternal age has little effect on offspring number and quality in Cardiocondyla ants
Source: Ecol Evol. 2018 Nov 8;8(23):12066–72. doi: 10.1002/ece3.4666 (PMC6303694; doi:10.1002/ece3.4666)
Supplement: Supplementary file 2 [file ECE3-8-12066-s002.docx]

**Results of GLMs and GLMMs with details on data transformation and models.**

**Mean egg production (N=25)**

Data transformation: No transformation

Model: linear model (lm function in R)

(1) mean_egg_production ~ queen_age + male_age + queen_age : male_age

Anova Table (Type III tests)

Response: mean_egg_production

Sum Sq Df F value Pr(>F)

(Intercept) 464.40 1 53.8588 3.19e-07 ***

queen_age 2.78 1 0.3226 0.5761

male_age 3.59 1 0.4168 0.5255

queen_age:male_age 5.60 1 0.6498 0.4292

Residuals 181.07 21

(2) mean_egg_production ~ queen_age + male_age

Anova Table (Type III tests)

Response: mean_egg_production

Sum Sq Df F value Pr(>F)

(Intercept) 600.62 1 70.7828 2.533e-08 ***

queen_age 14.93 1 1.7597 0.1983

male_age 0.08 1 0.0097 0.9224

Residuals 186.68 22

(3) mean_egg_production ~ queen_age

Anova Table (Type III tests)

Response: mean_egg_production

Sum Sq Df F value Pr(>F)

(Intercept) 737.86 1 90.8701 1.872e-09 ***

queen_age 14.87 1 1.8316 0.1891

Residuals 186.76 23

**Total sex offspring (N=25)**

Data transformation: No transformation

Model: generalized linear model (negative binomial model, glm.nb function in R)

(1) total_sex_offspring ~ queen_age + male_age + queen_age : male_age

Analysis of Deviance Table (Type III tests)

Response: total_sex_offspriong

LR Chisq Df Pr(>Chisq)

queen_age 0.80268 1 0.3703

male_age 0.82322 1 0.3642

queen_age:male_age 0.07508 1 0.7841

(2) total_sex_offspring ~ queen_age + male_age

Analysis of Deviance Table (Type III tests)

Response: total_sex_offspriong

LR Chisq Df Pr(>Chisq)

queen_age 0.83923 1 0.3596

male_age 1.08373 1 0.2979

(3) total_sex_offspring ~ male_age

Analysis of Deviance Table (Type III tests)

Response: total_sex_offspring

LR Chisq Df Pr(>Chisq)

male_age 1.4442 1 0.2295

**Sex ratio (N=17)**

Data transformation: $X^{3}$

Model: linear model (lm function in R)

(1) sex_ratio ~ queen_age + male_age + queen_age : male_age

Anova Table (Type III tests)

Response: (sex_ratio^3)

Sum Sq Df F value Pr(>F)

(Intercept) 1.24280 1 7.8589 0.01493 *

queen_age 0.14766 1 0.9337 0.35154

male_age 0.16204 1 1.0247 0.32989

queen_age:male_age 0.14295 1 0.9039 0.35907

Residuals 2.05582 13

(2) sex_ratio ~ queen_age + male_age

Anova Table (Type III tests)

Response: (sex_ratio^3)

Sum Sq Df F value Pr(>F)

(Intercept) 1.70511 1 10.8568 0.005315 **

queen_age 0.04372 1 0.2783 0.606050

male_age 0.04639 1 0.2954 0.595349

Residuals 2.19877 14

(3) sex_ratio ~ male_age

Anova Table (Type III tests)

Response: (sex_ratio^3)

Sum Sq Df F value Pr(>F)

(Intercept) 2.6800 1 17.9265 0.0007217 ***

male_age 0.0525 1 0.3512 0.5622671

Residuals 2.2425 15

**Queen bias (N=25)**

Data transformation: $\sqrt{X}$

Model: linear model (lm function in R)

(1) queen_bias ~ queen_age + male_age + queen_age : male_age

Anova Table (Type III tests)

Response: sqrt(queen_bias)

Sum Sq Df F value Pr(>F)

(Intercept) 0.65872 1 13.9819 0.00121 **

queen_age 0.06718 1 1.4260 0.24574

male_age 0.04027 1 0.8547 0.36573

queen_age:male_age 0.03288 1 0.6978 0.41291

Residuals 0.98937 21

(2) queen_bias ~ queen_age + male_age

Anova Table (Type III tests)

Response: sqrt(queen_bias)

Sum Sq Df F value Pr(>F)

(Intercept) 0.64949 1 13.9779 0.001138 **

queen_age 0.03533 1 0.7604 0.392625

male_age 0.01029 1 0.2215 0.642518

Residuals 1.02224 22

(3) queen_bias ~ queen_age

Anova Table (Type III tests)

Response: sqrt(queen_bias)

Sum Sq Df F value Pr(>F)

(Intercept) 0.70840 1 15.7797 0.0006025 ***

queen_age 0.04031 1 0.8979 0.3531984

Residuals 1.03254 23

**Wing shedding (N=25)**

Data transformation: $\sqrt{25+X}$

Model: linear model (lm function in R)

(1) wing_shedding ~ queen_age + male_age + queen_age : male_age

Anova Table (Type III tests)

Response: sqrt(25 + wing_shedding)

Sum Sq Df F value Pr(>F)

(Intercept) 372.24 1 404.5725 3.348e-15 ***

queen_age 33.11 1 35.9898 5.922e-06 ***

male_age 0.00 1 0.0039 0.9507

queen_age:male_age 1.39 1 1.5115 0.2325

Residuals 19.32 21

(2) wing_shedding ~ queen_age + male_age

Anova Table (Type III tests)

Response: sqrt(25 + wing_shedding)

Sum Sq Df F value Pr(>F)

(Intercept) 420.80 1 446.9557 4.164e-16 ***

queen_age 39.83 1 42.3102 1.525e-06 ***

male_age 1.26 1 1.3424 0.259

Residuals 20.71 22

(3) wing_shedding ~ queen_age

Anova Table (Type III tests)

Response: sqrt(25 + wing_shedding)

Sum Sq Df F value Pr(>F)

(Intercept) 535.83 1 560.784 < 2.2e-16 ***

queen_age 38.74 1 40.544 1.695e-06 ***

Residuals 21.98 23

**Start egg production (N=25)**

Data transformation: $\log X$

Model: linear model (lm function in R)

(1) start_egg_production ~ queen_age + male_age + queen_age : male_age

Anova Table (Type III tests)

Response: log(start_egg_production)

Sum Sq Df F value Pr(>F)

(Intercept) 71.034 1 200.4044 3.267e-12 ***

queen_age 0.875 1 2.4694 0.1310

male_age 0.596 1 1.6801 0.2090

queen_age:male_age 0.029 1 0.0819 0.7775

Residuals 7.443 21

(2) start_egg_production ~ queen_age + male_age

Anova Table (Type III tests)

Response: log(start_egg_production)

Sum Sq Df F value Pr(>F)

(Intercept) 82.997 1 244.3524 2.136e-13 ***

queen_age 1.086 1 3.1974 0.08754 .

male_age 0.867 1 2.5511 0.12448

Residuals 7.473 22

(3) start_egg_production ~ queen_age

Anova Table (Type III tests)

Response: log(start_egg_production)

Sum Sq Df F value Pr(>F)

(Intercept) 92.446 1 254.9780 6.128e-14 ***

queen_age 1.334 1 3.6804 0.06755 .

Residuals 8.339 23

**Hatchability (N=25)**

Data transformation: No transformation

Model: linear model (lm function in R)

(1) hatchability ~ queen_age + male_age + queen_age : male_age

Anova Table (Type III tests)

Response: hatchability

Sum Sq Df F value Pr(>F)

(Intercept) 2.93287 1 38.4622 3.752e-06 ***

queen_age 0.00007 1 0.0010 0.9755

male_age 0.18369 1 2.4090 0.1356

queen_age:male_age 0.01291 1 0.1694 0.6848

Residuals 1.60132 21

(2) hatchability ~ queen_age + male_age

Anova Table (Type III tests)

Response: hatchability

Sum Sq Df F value Pr(>F)

(Intercept) 3.3013 1 44.9931 9.64e-07 ***

queen_age 0.0092 1 0.1258 0.72616

male_age 0.2475 1 3.3734 0.07981

Residuals 1.6142 22

(3) hatchability ~ male_age

Anova Table (Type III tests)

Response: hatchability

Sum Sq Df F value Pr(>F)

(Intercept) 5.2486 1 74.3575 1.157e-08 ***

male_age 0.2399 1 3.3984 0.07818

Residuals 1.6235 23

**Thorax_length (N = 63)**

Data transformation: No transformation

Model: linear mixed model (lmer function in R, colony as a random factor)

(1) thorax_length ~ queen_age + male_age + queen_age : male_age + (1|colony)

Analysis of Deviance Table (Type III Wald chisquare tests)

Response: thorax_length

Chisq Df Pr(>Chisq)

(Intercept) 14487.5946 1 < 2e-16 ***

queen_age 5.6689 1 0.01727 *

male_age 2.3433 1 0.12582

queen_age:male_age 3.8215 1 0.05060 .

(2) thorax_length ~ queen_age + male_age + (1|colony)

Analysis of Deviance Table (Type III Wald chisquare tests)

Response: thorax_length

Chisq Df Pr(>Chisq)

(Intercept) 16284.4410 1 <2e-16 ***

queen_age 2.1813 1 0.1397

male_age 0.0495 1 0.8239

(3) thorax_length ~ male_age + (1|colony)

Analysis of Deviance Table (Type III Wald chisquare tests)

Response: thorax_length

Chisq Df Pr(>Chisq)

(Intercept) 22815.0332 1 <2e-16 ***

male_age 0.1964 1 0.6576

**Head_width (N = 63)**

Data transformation: $X^{3}$

Model: linear mixed model (lmer function in R, colony as a random factor)

(1) head_width ~ queen_age + male_age + queen_age : male_age + (1|colony)

Analysis of Deviance Table (Type III Wald chisquare tests)

Response: (head_width^3)

Chisq Df Pr(>Chisq)

(Intercept) 2231.5875 1 < 2e-16 ***

queen_age 0.0049 1 0.94395

male_age 2.7846 1 0.09517 .

queen_age:male_age 0.9001 1 0.34276

(2) head_width ~ queen_age + male_age + (1|colony)

Analysis of Deviance Table (Type III Wald chisquare tests)

Response: (head_width^3)

Chisq Df Pr(>Chisq)

(Intercept) 2638.2207 1 <2e-16 ***

queen_age 0.6365 1 0.4250

male_age 1.9820 1 0.1592

(3) head_width ~ male_age + (1|colony)

Analysis of Deviance Table (Type III Wald chisquare tests)

Response: (head_width^3)

Chisq Df Pr(>Chisq)

(Intercept) 3681.0274 1 <2e-16 ***

male_age 1.7094 1 0.1911

**Fluctuating asymmetry (N = 63)**

Data transformation: $\log10+X$

Model: linear mixed model (lmer function in R, colony as a random factor)

(1) fluctuating_asymmetry ~ queen_age + male_age + queen_age : male_age + (1|colony)

Analysis of Deviance Table (Type III Wald chisquare tests)

Response: log(10 + fluctuating_asymmetry)

Chisq Df Pr(>Chisq)

(Intercept) 1042.8126 1 <2e-16 ***

queen_age 1.3147 1 0.2515

male_age 1.7014 1 0.1921

queen_age:male_age 2.6853 1 0.1013

(2) fluctuating_asymmetry ~ queen_age + male_age + (1|colony)

Analysis of Deviance Table (Type III Wald chisquare tests)

Response: log(10 + fluctuating_asymmetry)

Chisq Df Pr(>Chisq)

(Intercept) 1135.0136 1 <2e-16 ***

queen_age 0.0414 1 0.8387

male_age 0.0473 1 0.8278

(3) fluctuating_asymmetry ~ male_age + (1|colony)

Analysis of Deviance Table (Type III Wald chisquare tests)

Response: log(10 + fluctuating_asymmetry)

Chisq Df Pr(>Chisq)

(Intercept) 1615.9035 1 <2e-16 ***

male_age 0.0638 1 0.8006

**Worker weight (N = 118)**

Data transformation: No transformation

Model: linear mixed model (lmer function in R, colony as a random factor)

(1) worker_weight ~ queen_age + male_age + queen_age : male_age + (1|colony)

Analysis of Deviance Table (Type III Wald chisquare tests)

Chisq Df Pr(>Chisq)

(Intercept) 1733.7651 1 < 2.2e-16 ***

queen_age 0.6778 1 0.410346

male_age 9.0679 1 0.002601 **

queen_age:male_age 2.1369 1 0.143789

(2) worker_weight ~ queen_age + male_age + (1|colony)

Analysis of Deviance Table (Type III Wald chisquare tests)

Chisq Df Pr(>Chisq)

(Intercept) 2017.6720 1 < 2.2e-16 ***

queen_age 0.0076 1 0.930363

male_age 7.4430 1 0.006368 **

(3) worker_weight ~ male_age + (1|colony)

Analysis of Deviance Table (Type III Wald chisquare tests)

Chisq Df Pr(>Chisq)

(Intercept) 2673.6597 1 < 2.2e-16 ***

male_age 7.6036 1 0.005825 **

**Female sexual weight (N=100)**

Data transformation: $X^{3.5}$

Model: linear mixed model (lmer function in R, colony as a random factor)

(1) female sexual_weight ~ queen_age + male_age + queen_age : male_age + (1|colony)

Analysis of Deviance Table (Type III Wald chisquare tests)

Response: (gyne_weight^3.5)

Chisq Df Pr(>Chisq)

(Intercept) 129.0943 1 < 2.2e-16 ***

queen_age 2.1928 1 0.1386597

male_age 11.8671 1 0.0005713 ***

queen_age:male_age 2.5219 1 0.1122728

(2) female sexual_weight ~ queen_age + male_age + (1|colony)

Analysis of Deviance Table (Type III Wald chisquare tests)

Response: (gyne_weight^3.5)

Chisq Df Pr(>Chisq)

(Intercept) 172.7623 1 < 2.2e-16 ***

queen_age 0.2065 1 0.649489

male_age 9.6494 1 0.001894 **

(3) female sexual_weight ~ male_age + (1|colony)

Analysis of Deviance Table (Type III Wald chisquare tests)

Response: (gyne_weight^3.5)

Chisq Df Pr(>Chisq)

(Intercept) 213.417 1 < 2.2e-16 ***

male_age 11.103 1 0.0008619 ***
