# Supplementary material for: Methuselah’s daughters: Paternal age has little effect on offspring number and quality in Cardiocondyla ants
Source: Ecol Evol. 2018 Nov 8;8(23):12066–72. doi: 10.1002/ece3.4666 (PMC6303694; doi:10.1002/ece3.4666)
Supplement: Supplementary file 3 [file ECE3-8-12066-s003.docx]

| **Results of Mann-Whitney U-tests about the influence of age on traits of queens and their offspring.** | | | | | | | |
| --- | --- | --- | --- | --- | --- | --- | --- |
| **queen age** | **median, quartiles**  **young** | **median, quartiles**  **old** | ***n_1_*** | ***n_2_*** | ***U*** | ***p exact*** | ***d_Cohen_*** |
| **queen traits** |  |  |  |  |  |  |  |
| mean egg production | 7.21; 5.63; 9.03 | 5.92; 5.04; 7.72 | 13 | 12 | 58 | 0.295 | 0.446 |
| total sexuals | 8; 3; 24 | 0.5; 0; 11 | 13 | 12 | 46 | 0.087 | 0.743 |
| sex ratio | 0.79; 0.27; 0.94 | 0.86; 0.70; 0.95 | 11 | 6 | 29 | 0.733 | 0.196 |
| queen bias | 0.06; 0; 0.12 | 0; 0; 0.10 | 13 | 12 | 62 | 0.406 | 0.354 |
| wing shedding (days after colony set-up) | 13; 10; 20 | -9.5; -15.25; -5.75 | 13 | 12 | 9.5 | 0.00004 | 2.235 |
| start of egg production (days after colony set-up) | 12; 10; 27 | 8; 5.5; 16.5 | 13 | 12 | 44.5 | 0.068 | 0.783 |
| egg hatchability | 0.47; 0.36; 0.73 | 0.47; 0.30; 0.69 | 13 | 12 | 75 | 0.894 | 0.065 |
| **offspring traits** |  |  |  |  |  |  |  |
| worker thorax length (mm) | 0.51; 0.50; 0.53 | 0.52; 0.51; 0.54 | 35 | 28 | 389 | 0.166 | 0.358 |
| worker head width (mm) | 0.41; 0.40; 0.42 | 0.41, 0.40; 0.42 | 35 | 28 | 483 | 0.929 | 0.024 |
| eye fluctuating asymmetry (µm] | 15.0; 5.6; 21.6 | 13.8; 6.1; 22.6 | 35 | 28 | 482.5 | 0.918 | 0.026 |
| worker weight (mg) | 0.121; 0.114; 0.132 | 0.119; 0.111; 0.137 | 64 | 54 | 1667.5 | 0.745 | 0.060 |
| female sexual weight (mg) | 0.201; 0.187; 0.213 | 0.198; 0.186; 0.212 | 77 | 23 | 816 | 0.574 | 0.114 |
| **male age** |  |  |  |  |  |  |  |
| **queen traits** |  |  |  |  |  |  |  |
| mean egg production | 7.02; 5.38; 8.81 | 6; 5.63; 7.54 | 16 | 9 | 68 | 0.846 | 0.091 |
| total sexuals | 4.5; 0.75; 24 | 3; 0; 10 | 16 | 9 | 54.5 | 0.329 | 0.404 |
| sex ratio | 0.83; 0.39; 0.92 | 0.86; 0.70; 1.00 | 12 | 5 | 25 | 0.646 | 0.258 |
| queen bias | 0.09; 0.03; 0.12 | 0; 0; 0.13 | 16 | 9 | 64 | 0.677 | 0.182 |
| wing shedding (days after colony set-up) | 7.5; -10; 19 | 6; -7; 12 | 16 | 9 | 71 | 0.978 | 0.023 |
| start of egg production (days after colony set-up) | 15.5; 8; 23.25 | 9; 6; 11 | 16 | 9 | 42 | 0.095 | 0.722 |
| egg hatchability | 0.55; 0.42; 0.80 | 0.31; 0.26; 0.47 | 16 | 9 | 38 | 0.057 | 0.834 |
| **offspring traits** |  |  |  |  |  |  |  |
| worker thorax length (mm) | 0.52; 0.50; 0.53 | 0.41; 0.40; 0.42 | 41 | 22 | 431 | 0.773 | 0.073 |
| worker head width (mm) | 0.52; 0.51; 0.53 | 0.41; 0.41; 0.42 | 41 | 22 | 352.5 | 0.155 | 0.364 |
| eye fluctuating asymmetry (µm] | 15.0; 5.7; 23.0 | 14.2; 6.5; 21.6 | 41 | 22 | 432 | 0.784 | 0.069 |
| worker weight (mg) | 0.122; 0.115; 0.139 | 0.116; 0.111; 0.127 | 75 | 43 | 1148 | 0.009 | 0.493 |
| female sexual weight (mg) | 0.203; 0.191; 0.216 | 0.185; 0.180; 0.196 | 83 | 17 | 362 | 0.001 | 0.664 |
